# Patients with abdominal aortic aneurysms have reduced levels of microRNA 122-5p in circulating exosomes

**DOI:** 10.1371/journal.pone.0281371

**Published:** 2023-02-14

**Authors:** Jose L. Lopez, Joel L. Ramirez, Tuan Anh Phu, Phat Duong, Laura Bouchareychas, Christina R. Kuhrau, Pei-Yu Lin, Walter L. Eckalbar, Andrea J. Barczak, Joshua D. Rudolph, Lenka Maliskova, Michael S. Conte, Shant M. Vartanian, Robert L. Raffai, Adam Z. Oskowitz

**Affiliations:** 1 Division of Vascular and Endovascular Surgery, Department of Surgery, University of California San Francisco, San Francisco, California, United States of America; 2 Chan Zuckerberg Biohub, San Francisco, California, United States of America; 3 Department of Veterans Affairs, Surgical Service (112G), San Francisco, California, United States of America; 4 Division of Pulmonary and Critical Care Medicine, Department of Medicine, UCSF CoLabs, University of California San Francisco, San Francisco, California, United States of America; University of Missouri School of Medicine, UNITED STATES

## Abstract

**Objective:**

There are currently no specific biomarkers to identify patients with abdominal aortic aneurysms (AAAs). Circulating exosomes contain microRNAs (miRNA) that are potential biomarkers for the presence of disease. This study aimed to characterize the exosomal miRNA expression profile of patients with AAAs in order to identify novel biomarkers of disease.

**Methods:**

Patients undergoing duplex ultrasound (US) or computed tomography (CT) for screening or surveillance of an AAA were screened to participate in the study. Cases with AAA were defined as having a max aortic diameter >3 cm. Circulating plasma exosomes were isolated using Cushioned-Density Gradient Ultracentrifugation and total RNA was extracted. Next Generation Sequencing was performed on the Illumina HiSeq4000 SE50. Differential miRNA expression analysis was performed using DESeq2 software with a Benjamini-Hochberg correction. MicroRNA expression profiles were validated by Quantitative Real-Time PCR.

**Results:**

A total of 109 patients were screened to participate in the study. Eleven patients with AAA and 15 non-aneurysmal controls met study criteria and were enrolled. Ultrasound measured aortic diameter was significantly larger in the AAA group (mean maximum diameter 4.3 vs 2.0 cm, *P* = 6.45x10^-6^). More AAA patients had coronary artery disease (5/11 vs 1/15, *P* = 0.05) as compared to controls, but the groups did not differ significantly in the rates of peripheral arterial disease and chronic obstructive pulmonary disease. A total of 40 miRNAs were differentially expressed (*P*<0.05). Of these, 18 miRNAs were downregulated and 22 were upregulated in the AAA group compared to controls. After false discovery rate (FDR) adjustment, only miR-122-5p was expressed at significantly different levels in the AAA group compared to controls (fold change = 5.03 controls vs AAA; raw *P* = 1.8x10^-5^; FDR *P* = 0.02).

**Conclusion:**

Plasma exosomes from AAA patients have significantly reduced levels of miRNA-122-5p compared to controls. This is a novel exosome-associated miRNA that warrants further investigation to determine its use as a diagnostic biomarker and potential implications in AAA pathogenesis.

## Introduction

Abdominal aortic aneurysms (AAAs) are the 15^th^ leading cause of death in the United States and undergo slow but sustained growth, leaving a long interval between potential diagnosis and the need for treatment [1–3]. The risk of rupture increases with size and surgical intervention is recommended when the risk of aneurysm rupture exceeds that of surgery, which is most commonly due to aneurysm size (5.5 cm in men and >5.0 cm in women) [1]. Early diagnosis through screening is essential to minimize morbidity and mortality from the disease. The current recognized screening method for AAA is a duplex ultrasound (US) of the aorta [4]. The United States Preventative Service Task Force (USPSTF) recommends a one-time screening for AAA with US in men ages 65–75 who have ever smoked [4]. However, this procedure is relatively time consuming, costly, and may not be easily available to everyone [5–8]. The prevalence of AAA is estimated to be 2.2% in the United States, suggesting there are over a million people affected by the disease, far exceeding the current screening capacity [9]. Furthermore, duplex US and the training programs needed to perform these exams are not readily available throughout the world [10–13]. Accordingly, identifying a biomarker to efficiently screen and perform surveillance for AAA would have a substantial worldwide impact.

Circulating exosomes have been identified as biomarkers for numerous diseases, including several cardiovascular diseases [14–16]. These nanometer-sized vesicles are released from various cell types and circulate within the bloodstream, delivering their contents to distant sites and other cells within the body. Exosomes contain an array of effector molecules including proteins, lipids, and RNA [17, 18]. In particular, a subset of small non-coding RNAs, known as microRNAs (miRNA) are concentrated within exosomes [17]. MicroRNAs regulate gene expression through post-transcriptional degradation of messenger RNA (mRNA), inhibiting translation [19]. These molecules have been described as playing an important role in regulating key components of aneurysm formation, including macrophage and T-cell function, metalloproteinase activity and endothelial cell homeostasis [20–23]. Given the role they play in regulating processes related to AAA formation and growth, miRNA within circulating exosomes could serve to help identify those at risk for AAA development. The aim of this study was to characterize the exosomal miRNA expression profile of patients with AAA in order to identify novel biomarkers of disease.

## Material and methods

### Patient recruitment

This study was approved by the University of California, San Francisco (UCSF) Institutional Review Board (18–26351). Patients ≥ 55 years of age were recruited at the UCSF Vascular Surgery outpatient clinics and vascular laboratories between January 2019 and 2020. All patients who underwent screening or surveillance imaging for AAA during this time were reviewed for possible study participation based on the inclusion and exclusion criteria stated below [4]. Cases were defined as patients with a maximum infrarenal abdominal aorta diameter of ≥ 3 cm on US or computed tomography (CT). Controls, or patients without aneurysms, were defined as having a maximum infrarenal abdominal aorta diameter of < 3 cm on US or CT. Patients were excluded from participating if they had severe hepatic (Child-Pugh ≥ B) or renal (creatinine ≥ 2 mg/dL) dysfunction, a non-vascular inflammatory disease, a severe acute illness or surgery within 30 days, or were taking immunosuppressive medications or steroids (Table 1). The inclusion and exclusion criteria were selected with the goal of reducing the possible influence of acute and chronic diseases that may directly affect circulating miRNA patterns, with a focus on diseases associated with changes in systemic inflammation.

**Table 1:** Study inclusion and exclusion criteria.

Inclusion	Exclusion
≥ 55 years of age	Evidence of active infection
Duplex US or CT of the aorta within the last 6 months	Chronic liver disease (Child-Pugh ≥ B)
Provision of informed consent for blood sample storage and analysis	End-stage renal disease (CKD 5 or creatinine ≥ 2 mg/dL)
	Chronic inflammatory disorders
	BMI < 20 or >35
	Major surgery or illness within 30 days
	Use of immunosuppressive medications or steroids
	History of organ transplantation
	Pregnancy or lactating

US, ultrasound; CT, computed tomography; CKD, chronic kidney disease; BMI, body mass index.

### Patient data and imaging

Patients who met the appropriate criteria were approached about the study during a clinic visit. After informed written consent was obtained from the study participants, they were interviewed in-person and a questionnaire was used to collect demographic variables, medical comorbidities, smoking history, and medication use. Participant medical records were cross-referenced to ensure accuracy of data. A blood sample was collected from participants at that time and processed as described below. Aneurysm measurements were obtained from either US or CT based on the evaluation of a board-certified Diagnostic Radiology or board-certified and Registered Physician in Vascular Interpretation (RPVI)-certified Vascular Surgeon.

### Sample collection, plasma isolation, and cytokine measurement

Venipuncture and blood sample collection were performed in accordance with the policies of the UCSF IRB. A total of four 10 mL peripheral venous blood samples (3-EDTA and 1-serum separator tube) were collected from each participant at the time of enrollment. The samples were transported to our laboratory at room temperature and centrifuged at 2000 x g for 15 minutes to separate the plasma. The resultant plasma fraction was carefully aspirated and 1mL of plasma was aliquoted to each sterile cryogenic vial. Plasma samples were frozen at -80°C until exosome isolation and RNA extraction. All samples were processed within 2 hours of collection. Plasma levels of interleukin-6 (IL-6) were measured using a commercially available enzyme-linked immunosorbent assay (ELISA) kit from Sigma-Aldrich according to the manufacturer’s instructions (Sigma-Aldrich, St. Louis, MO).

### Plasma exosomes isolation and characterization

Plasma exosomes were isolated and characterized according to methods recently reported by our group [24, 25]. Briefly, 0.9 mL of plasma was centrifuged at 1,500 x g for 10 minutes at 4°C and diluted in 38.5 mL of cold phosphate buffered saline. The diluted plasma was then transferred to a Quick-Seal Ultra-Clear centrifuge tube (Beckman Coulter, Indianapolis, IN) and underlaid with 2 mL of 60% OptiPrep iodixanol cushion (Sigma-Aldrich, St. Louis, MO) at 100,000 x g for 3 hours at 4°C (Type 50.2 Ti Rotor, Beckman Coulter). The bottom 3 mL was then collected and underlaid with a step density gradient (5%, 10%, 20% w/v OptiPrep iodixanol) in an Ultra-Clear centrifuge tube (Beckman Coulter, Indianapolis, IN). The density gradient was subsequently spun at 100,000 x g for 18 hours at 4°C (SW 40 Ti Rotor, Beckman Coulter, Indianapolis, IN) [24]. Subsequently, twelve 1 mL fractions were collected starting from the top of the tube. NanoSight LM14 (Malvern Instruments, Westborough, MA) was used to measure (EV) size and concentration in Fraction 7 using a 488-nm detection wavelength. The analysis settings were optimized and kept identical for each sample, with a detection threshold set at 3; three videos of 1 min each were analyzed to give the mean, mode, median, and estimated concentration for each particle size. Samples were diluted in 1:50 in PBS and measured in triplicate. Data was analyzed with the NTA 3.2 software.

For western blot analysis, the EV fraction was mixed with Laemmli buffer (Bio-Rad, Hercules, CA) and boiled at 95°C for 5 minutes in non-reducing conditions. Samples were then loaded on a 10% SDS-PAGE gel and transferred onto PVDF membrane (Bio-Rad, Hercules, CA). The membranes were blocked with 5% non-fat milk dissolved in PBS for one hour and then were probed with primary antibodies overnight at 4°C primary antibodies: anti-CD81 (1:200, Abcam, Cambridge, Mass), anti-CD63 (1:200, BD Pharmingen, San Jose, CA). After 4 washes in PBS containing 0.1% Tween (PBST), membranes were incubated with anti-mouse IgG-HRP (1:1000, Santa Cruz Biotechnology, Dallas, TX) for 1h and washed in PBST. Signals were visualized after incubation with Amersham ECL Prime substrate (Sigma-Aldrich, St. Louis, MO) and imaged using an ImageQuant LAS 4000 (GE healthcare, Chicago, IL).

### Exosome total RNA extraction

Total RNA was extracted from Fraction 7 using a Plasma/Serum Circulating and Exosomal RNA Purification Kit (Slurry Format; Norgen Biotek Corp., Thorold, ON, Canada) according to the manufacturer’s instructions. The RNA concentration was measured with Quant-iT Ribogreen RNA Assay (Thermo Fisher, Waltham, Mass).

### Small RNA library preparation and next-generation sequencing

Exosomal RNA was evaporated and concentrated completely via SpeedVac and resuspended in 6uL of RNase/DNase free water. Small RNA libraries were prepared using the NEBNext Mulitplex Small RNA Library Prep Set for Illumina (cat#E7300, New England Biolabs, Ipswich, MA) according to the manufacturer’s protocol with 24 cycles for library amplification. Briefly, 5’ and 3’ adapters were ligated with exosomal RNA samples, followed by cDNA library construction. Libraries were purified with Zymo DNA Clean & Concentrator according to protocol and normalized according to Nanodrop readings. The libraries were pooled, and size was selected for 132bp to 150bp on Blue Pippin Automated DNA Size Selection (Sage Science, Beverly, MA). Library quality control was performed on a MiniSeq Sequencing System (Illumina, San Diego, CA) and the libraries were then sequenced on 3 lanes of a HiSeq4000 SE50 (Illumina, San Diego, CA).

### Sequencing data and miRNA differential expression analysis

The raw data from the HiSeq4000 SE50 (Illumina, San Diego, CA) was assessed for quality with FastQC software (Babraham Bioinformatics, Cambridge, UK) [26]. The sequence reads were trimmed to remove adapters and low-quality bases and reads less than 18 nucleotides were discarded. Files were exported to Genboree Workbench (Bioinformatics Research Laboratory, Houston, Texas, version 4.6.3) for read alignment to the human GRCh38 genome (Ensembl, Hinxton, UK). Reads were first mapped to UniVec and human ribosomal rRNA (rRNA) sequences to exclude them before mapping to miRbase (Manchester, UK, version 21, http://mirbase.org/) [27]. The expression of miRNAs was normalized as reads per million (RPM). Differential expression analysis was performed with DESeq2 (Bioconductor, version 1.26.0) using a Wald test for statistical significance (p <0.05) [28, 29]. A Benjamini-Hochberg correction for multiple comparisons was used with a false discovery rate (FDR) p <0.05 to identify statistically significant changes in expression.

### Real-Time qPCR validation of miR-122 expression profile

Reverse transcription was performed using the miRCURY LNA Universal RT miRNA Kit (Qiagen, Germantown, MD) according to the manufacturer’s instructions. Briefly, 4uL of exosomal RNA was polyadenylated and converted to cDNA. A total of 0.5uL of UniSp6 RNA was spiked in the RT mix for cDNA synthesis quality control. Total reaction volume was 10uL. Reaction temperature cycling consisted of an RT step at 42°C for 60 minutes, inactivation at 95°C for 5 minutes, and immediate cooling to 4°C. Real-time qPCR was performed using miRCURY LNA SYBR^®^ Green master mix and miRCURY LNA miRNA PCR Assay (Qiagen, Germantown, MD). The miRNA specific primers sets were as follows: hsa-miR-122-5p: 5’-UGGAGUGUGACAAUGGUGUUUG-3’, hsa-miR-16-5p: 5’ UAGCAGCACGUAAAUAUUGGCG-3’. All PCR assays were carried out on a QuantStudio 5 Flex Real-Time PCR System (Applied Biosystems. Inc, Foster City, CA) programmed as follows: 95°C for 2 min, followed by 40 cycles of 95°C for 10 s and 56°C for 1 min. Real-time qPCR assays (total reaction volume 10uL) for each target miRNA were set up in triplicate reactions. No-template controls were included. The comparative cycle threshold (CT) was used to evaluate the relative detection level of miR-122 in each sample and expression levels were normalized to miR-16-5p. Fold-change was calculated using the delta-delta CT method (2^−ΔΔCT^) of relative quantification.

### Statistical analysis

All statistical analyses were completed using an intention-to-treat method and performed using STATA (StataCorp, College Station, Texas, version 15.0). Summary statistics were reported using median and interquartile ranges (IQR) for continuous variables and frequency and percentage for categorical variables. Differences in characteristics between patients with and without aneurysm were calculated using a *X*^2^ test for categorical variables, a Fisher’s Exact text for parametric continuous variables, and a Wilcoxon Rank Sum Test for non-parametric continuous variables. Statistical significance was set at *P* ≤ 0.05. MicroRNA differential expression analysis was performed on DESeq2 (Bioconductor, version 1.26.0) [29]. Differentially expressed miRNAs were identified on the basis of a raw *P* <0.05 with statistical significance set at FDR <0.05. In qRT-PCR analysis, the mean relative expression of miR-122 between the two groups was compared using Student’s t-test with *P* <0.05. Given an FDR level of 0.05. maximum dispersion of 0.5, and a ratio of the geometric mean of normalization factors of 1, 10 participants would be required in each group to have 80% power to detect at least a fourfold change in a miRNA.

## Results

### Patient characteristics

**Fig 1:**
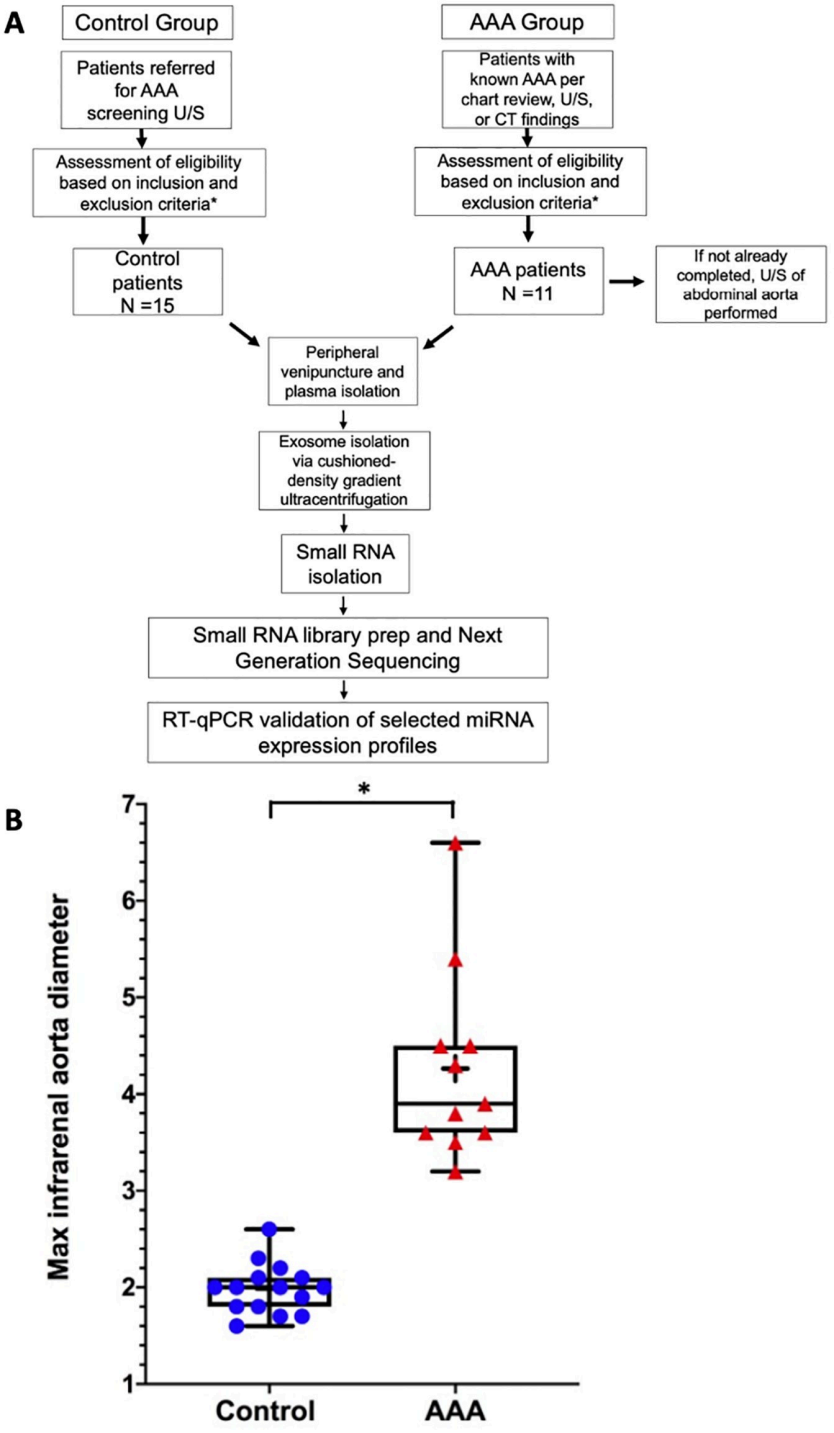
Flow diagram of patient selection and study design (**A**). Box and whisker plot showing maximum infrarenal aorta diameter of each patient enrolled in the study (**B**). AAA, abdominal aortic aneurysm; US, ultrasound; CT, computed tomography; qRT-PCR, quantitative real-time PCR.

A total of 109 patients were screened, 26 of whom were enrolled, 11 in the AAA group and 15 controls (Fig 1). Reasons for exclusion are displayed in Table 2. As shown in Table 3, more AAA patients than controls had coronary artery disease (45% vs 7%; *P* = .05) but the groups had similar levels of diabetes, peripheral artery disease (PAD), hypertension (HTN), and hyperlipidemia. More patients in the AAA group were taking aspirin (82% vs 33%; *P* = .02) and statins (100% vs 67%; *P* = .05). Infrarenal aorta diameter, as measured by US or CT, was significantly larger in the AAA group than in the control group (mean maximum diameter 4.3 vs 2.0 cm, *P* = 6.45x10^-6^) (Fig 1).

**Table 2:** Patients excluded from study.

	No Aneurysm	Aneurysm	^1^Unknown Aneurysm Status
**Exclusion Criteria**			
Active Infection	0	1	1
Chronic Liver, Renal, or Inflammatory Disease	2	3	7
Recent Major Surgery/Illness	0	2	0
Steroids or Immunosuppressive Medication	1	4	0
History of Organ Transplantation	0	1	0
Pregnancy or Lactating	0	0	0
^2^HIV/AIDS	0	1	3
^3^Age	1	2	0
^4^Communication Barrier	1	1	1
**Declined Participation**	9	5	0
^5^ **Screening Process Failure**	1	1	0
^6^ **Not Approached: Logistical**	3	17	15
^7^ **Total**	18	38	27

^1^. Patients excluded from the study before their AAA screening ultrasound results were obtained.

^2^. While not listed as a Table I Exclusion, HIV/AIDS was common enough to become a discrete category.

^3^. While not listed as a Table I Exclusion, 1 patient was < 55 years of age (did not meet inclusion criteria) and 2 elderly patients were considered too frail to draw blood from for non-medical purposes.

^4^. While not listed as a Table I Exclusion, 1 patient was not consented due to their mental status and 2 patients did not demonstrate enough comprehension of the study for the team to move forward.

^5^. Those who signed consent but were deemed ineligible after interviewing. Blood samples were not obtained from these patients.

^6^. Logistical problems included time constraints, lack of available diagnostic imaging, and patient no-shows.

^7^. Total does not reflect the exact number of patients screened. For example, several patients met multiple exclusions or met one exclusion and also lacked proper imaging.

AAA, abdominal aortic aneurysm; HIV, human immunodeficiency virus; AIDS, acquired immunodeficiency syndrome.

**Table 3:** Characteristics, medication use, and abdominal aorta size of patients enrolled in the study.

	No Aneurysm (n = 15)	Aneurysm (n = 11)	*P* value^a^
**Characteristic**			
Age (years)	67.0 ± 5.1	70.4 ± 6.3	0.15
Female	2 (13%)	1 (9%)	1.00
White	3 (20%)	6 (55%)	0.10
Stroke	1 (7%)	4 (36%)	0.13
Coronary Artery Disease	1 (7%)	5 (45%)	**0.05**
Congestive Heart Failure	0 (0%)	1 (9%)	0.42
Hypertension	14 (93%)	9 (82%)	0.56
Hyperlipidemia	12 (80%)	9 (82%)	1.00
Diabetes	3 (20%)	3 (27%)	1.00
Peripheral Artery Disease	2 (13%)	4 (36%)	0.35
COPD	4 (27%)	4 (36%)	0.68
Current Smoker	4 (27%)	3 (27%)	1.00
**Medication**			
Aspirin	5 (33%)	9 (82%)	**0.02**
Plavix	0 (0%)	1 (9%)	0.42
Anticoagulant	2 (13%)	2 (18%)	1.00
Beta blocker	6 (40%)	7 (64%)	0.43
Alpha blocker	0 (0%)	1 (9%)	0.42
ACE-I/ARB	10 (67%)	5 (45%)	0.43
Calcium channel blocker	4 (27%)	5 (45%)	0.42
Thiazide	7 (47%)	1 (9%)	0.08
Loop diuretic	0 (0%)	2 (18%)	0.17
Statin	10 (67%)	11 (100%)	**0.05**
Insulin	2 (13%)	0 (0%)	0.49
Metformin	3 (20%)	2 (18%)	1.00
**Abdominal Aorta (cm)**			
Proximal Diameter	2.0 ± 0.3	2.3 ± 0.3	**0.02**
Mid Diameter	1.6 ± 0.2	2.3 ± 0.5	**<0.01**
Distal Diameter	1.5 ± 0.2	4.2 ± 1.0	**<0.01**

Values as n (%) or mean ± standard deviation.

^a^Calculated using Student’s T-test for continuous variables and Fisher’s Exact test for categorical variables.

Total number of patients screened = 109.

COPD, chronic obstructive pulmonary disease; ACE-I, angiotensin-converting-enzyme inhibitors; ARB, angiotensin receptor blocker.

### Exosome characteristics

**Fig 2:**
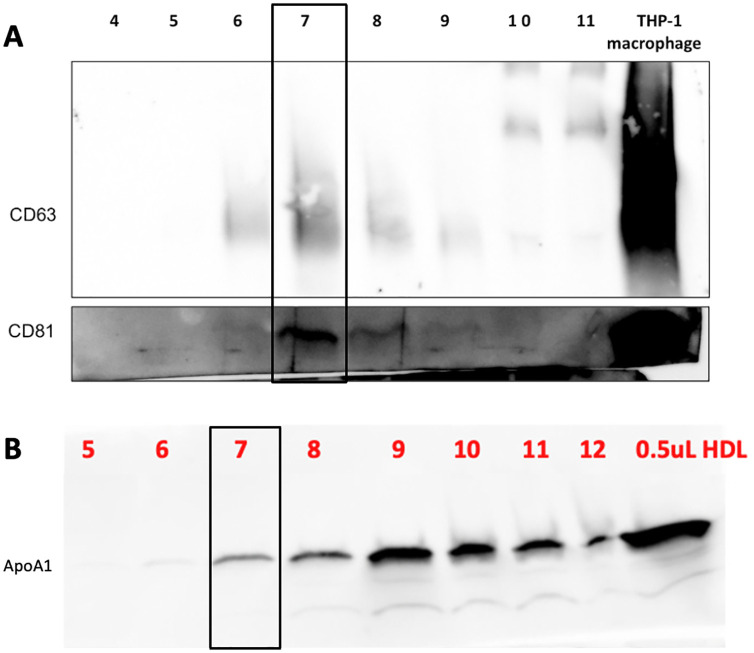
Characterization of plasma-derived exosomes. Representative Western blot indicating that exosomes expressed exosome specific markers—CD81, CD63, and CD9 and minimal expression of ApoA1, which is a marker of high-density lipid contamination. Cultured THP-1 macrophage exosomes were used as a positive control. Each column is numbered according to the resultant fraction from cushioned-density gradient ultracentrifugation.

Ultimately, only 24 patients (10 AAA and 14 control) had blood samples that were adequate for exosomal miRNA analyses. Nanoparticle tracking analysis revealed that Fraction 7’s average particle size in all of the samples ranged from 83.7 to 142.4 nanometers and the concentration of particles in this fraction ranged from 1.35x10^8^ to 5.33x10^8^. Western blot analysis showed that Fraction 7 had the highest concentration of exosome specific markers—CD81, CD63, and CD9 and minimal expression of ApoA1, which is a marker of high-density lipid contamination, as shown in Fig 2.

### Next generation sequencing data

#### Quality control and sequencing analysis

All 24 samples met quality control standards based on the number of transcriptome reads and the ratio of RNA-annotated reads to the genome read. Length distribution analysis revealed reads were in the range of 20–30 nucleotides (nt), which is consistent with the known length of miRNAs (S1 Fig). Sequencing resulted in 1.01 billion total reads with an average of 1.4% mapping to human miRNA. The range of input reads from each sample was from 1.78x10^7^ to 9.15x10^7^. The reads mapped to the human genome were classified based on the number normalized reads of various small RNA biotypes, among which protein coding and miRNA were the most represented (S1 Fig).

#### Differentially expressed miRNAs

**Fig 3:**
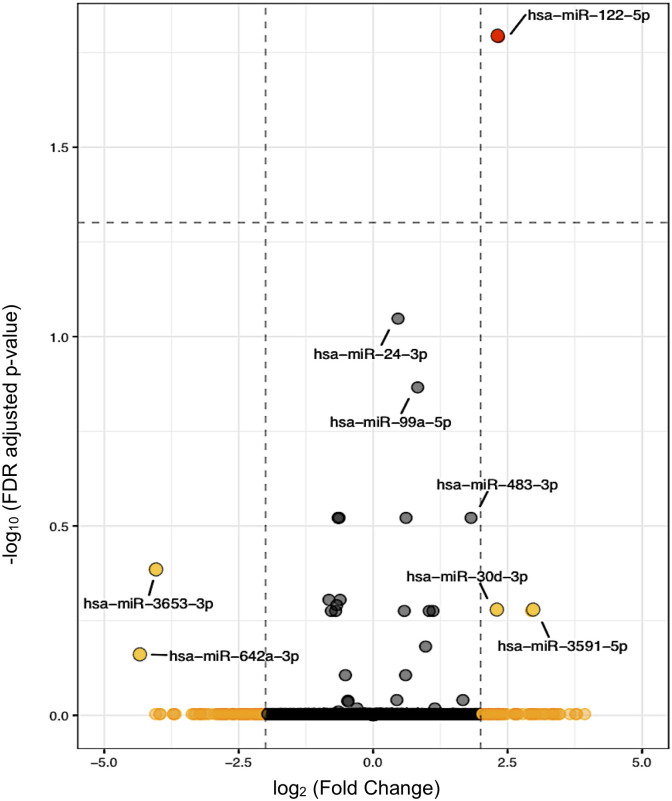
Volcano plot depicting miRNA differential expression in controls vs. AAA group. The dots represent the 40 miRNA that were differentially expressed as assessed by *P* < 0.05 before false discovery rate (FDR) adjustment. Dashed vertical lines indicate the threshold for a relative expression fold change (FC) of 2 or -2-fold. The dashed horizontal line represents the threshold of a 0.05 FDR value. The red dot (miRNA-122-5p) has an absolute log2 FC >2 and an FDR adjusted *P* < 0.05. The yellow dots represent miRNAs with an absolute log2 FC > 2 that were not statistically significant after FDR adjustment. FDR, false discovery rate.

Forty miRNAs were differentially expressed between the AAA and control groups (*P* <0.05): 18 were downregulated and 22 were upregulated (Table 4). The log_2_ fold change of the 40 differentially expressed miRNA indicated that miR-5100, miR-3591-5p, miR-122-5p, and miR-30d-3p were the most downregulated and miR-642a-3p, miR-3653-3p, and miR-4661-5p were the most upregulated genes in the AAA group compared to the control group (Fig 3). After a false discovery rate (FDR) adjustment was done, only miR-122-5p was found to be expressed at significantly different levels (control vs. AAA; |log_2_ fold change| > 2, FDR *P* = 0.02), demonstrating significantly reduced levels in AAA patients compared to controls. A network analysis using the miRabel miRNA database demonstrated that miR-122-5p has potential interactions with several MMPs, which are critical end targets in regulation of extracellular matrix breakdown in AAA. The two highest ranked MMPs identified were MMP7 (miRabel score 0.08) and MMP2 (miRabel score 0.46) [30].

**Table 4:** Exosomal miRNAs differentially expressed in AAA group vs. controls.

miRBase_ID	Fold Change	Description	*P* value^a^	FDR *P* Value^a^
hsa-miR-5100	0.065	Down	0.04941	0.993
hsa-miR-3591-5p	0.130	Down	0.01018	0.530
**hsa-miR-122-5p**	0.199	Down	**0.00002**	**0.016**
hsa-miR-30d-3p	0.203	Down	0.00695	0.526
hsa-miR-483-3p	0.283	Down	0.00160	0.301
hsa-miR-1468-5p	0.314	Down	0.02409	0.911
hsa-miR-192-5p	0.451	Down	0.02957	0.959
hsa-miR-195-3p	0.463	Down	0.01051	0.530
hsa-miR-27a-5p	0.485	Down	0.00775	0.530
hsa-miR-194-5p	0.508	Down	0.01377	0.658
hsa-miR-99a-5p	0.563	Down	0.00045	0.136
hsa-miR-24-2-5p	0.571	Down	0.04686	0.993
hsa-miR-125a-5p	0.654	Down	0.00194	0.301
hsa-let-7d-3p	0.658	Down	0.01898	0.784
hsa-miR-27a-3p	0.669	Down	0.00916	0.530
hsa-miR-24-3p	0.726	Down	0.00020	0.090
hsa-miR-126-3p	0.737	Down	0.02340	0.911
hsa-miR-27b-3p	0.776	Down	0.04632	0.993
hsa-let-7i-5p	1.230	Up	0.02955	0.959
hsa-let-7g-5p	1.289	Up	0.03328	0.993
hsa-let-7e-5p	1.350	Up	0.04860	0.993
hsa-miR-486-3p	1.383	Up	0.02636	0.920
hsa-miR-185-5p	1.383	Up	0.02519	0.915
hsa-miR-7-5p	1.429	Up	0.01836	0.784
hsa-miR-191-5p	1.447	Up	0.03549	0.993
hsa-miR-183-5p	1.464	Up	0.03748	0.993
hsa-miR-16-5p	1.531	Up	0.00546	0.496
hsa-let-7a-5p	1.553	Up	0.00232	0.301
hsa-miR-199a-3p|	1.563	Up	0.03118	0.976
hsa-miR-199b-3p				
hsa-let-7f-5p	1.570	Up	0.00223	0.301
hsa-miR-182-5p	1.600	Up	0.00620	0.512
hsa-let-7d-5p	1.623	Up	0.00841	0.530
hsa-miR-1827	1.712	Up	0.00953	0.530
hsa-miR-451a	1.767	Up	0.00510	0.496
hsa-miR-15b-5p	2.217	Up	0.03444	0.993
hsa-miR-3158-3p	2.519	Up	0.04678	0.993
hsa-miR-132-3p	3.846	Up	0.04341	0.993
hsa-miR-4661-5p	15.625	Up	0.04433	0.993
hsa-miR-3653-3p	16.667	Up	0.00362	0.411
hsa-miR-642a-3p	20.408	Up	0.01522	0.691

^a^Assessed by *P* < 0.05 and false-discovery rate (FDR) adjusted *P* < 0.05. Only miR-122 met significance after FDR adjustment.

^b^Upregulated genes shaded in green. Downregulated genes shaded in red. MicroRNA-122-5p shaded in yellow.

### Validation of miR-122 expression profile by qRT-PCR analysis

**Fig 4:**
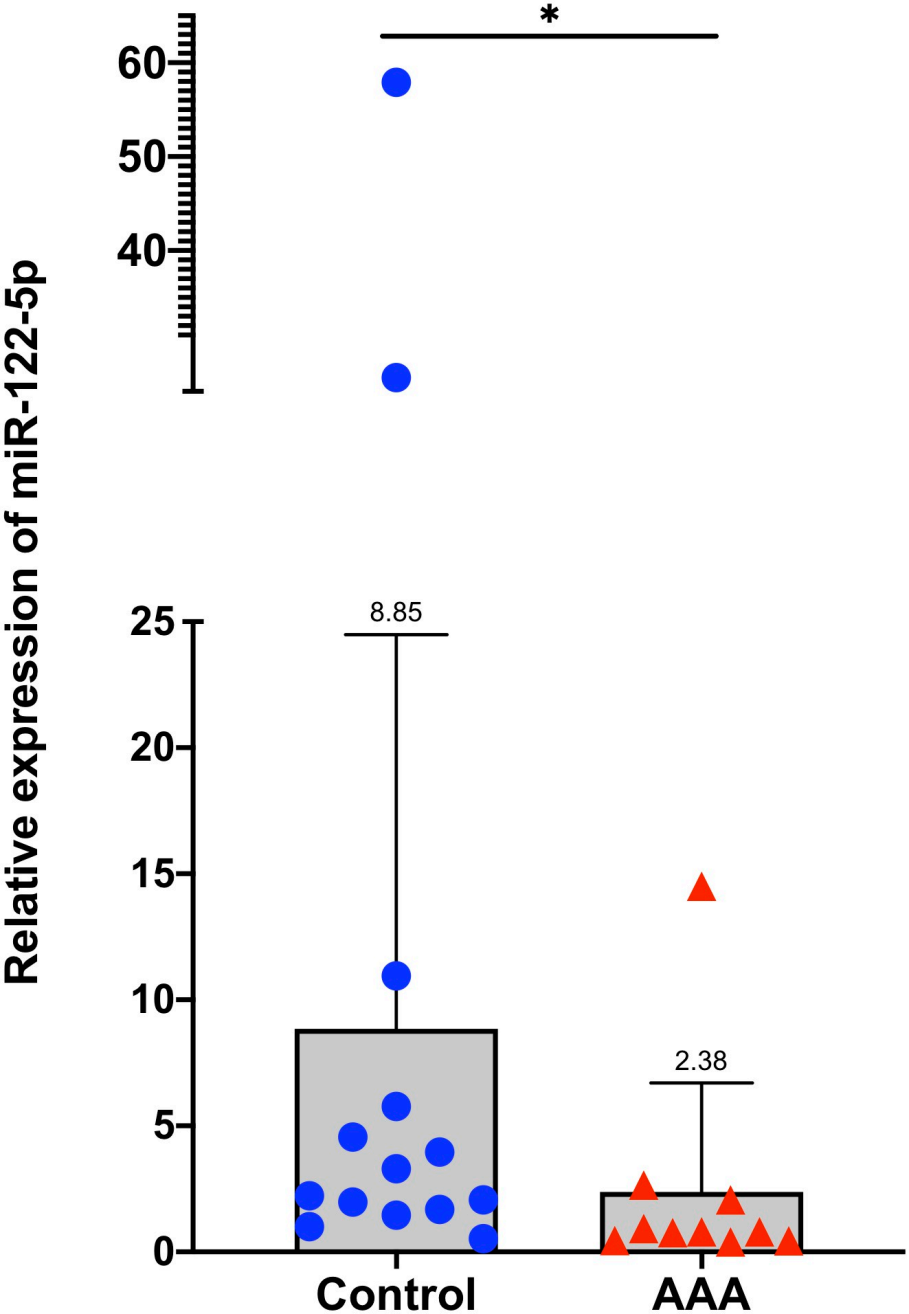
qRT-PCR analysis of miR-122-5p. MicroRNA levels are presented as fold changes (2-ΔΔCt). The Y-axis depicts values normalized to miR-16-5p. The mean relative expression of exosomal miR-122-5p was 2.38 vs. 8.85 in the AAA and control group, respectively (*P* = 0.026). *P <0.05. AAA, abdominal aortic aneurysm.

**Fig 5:**
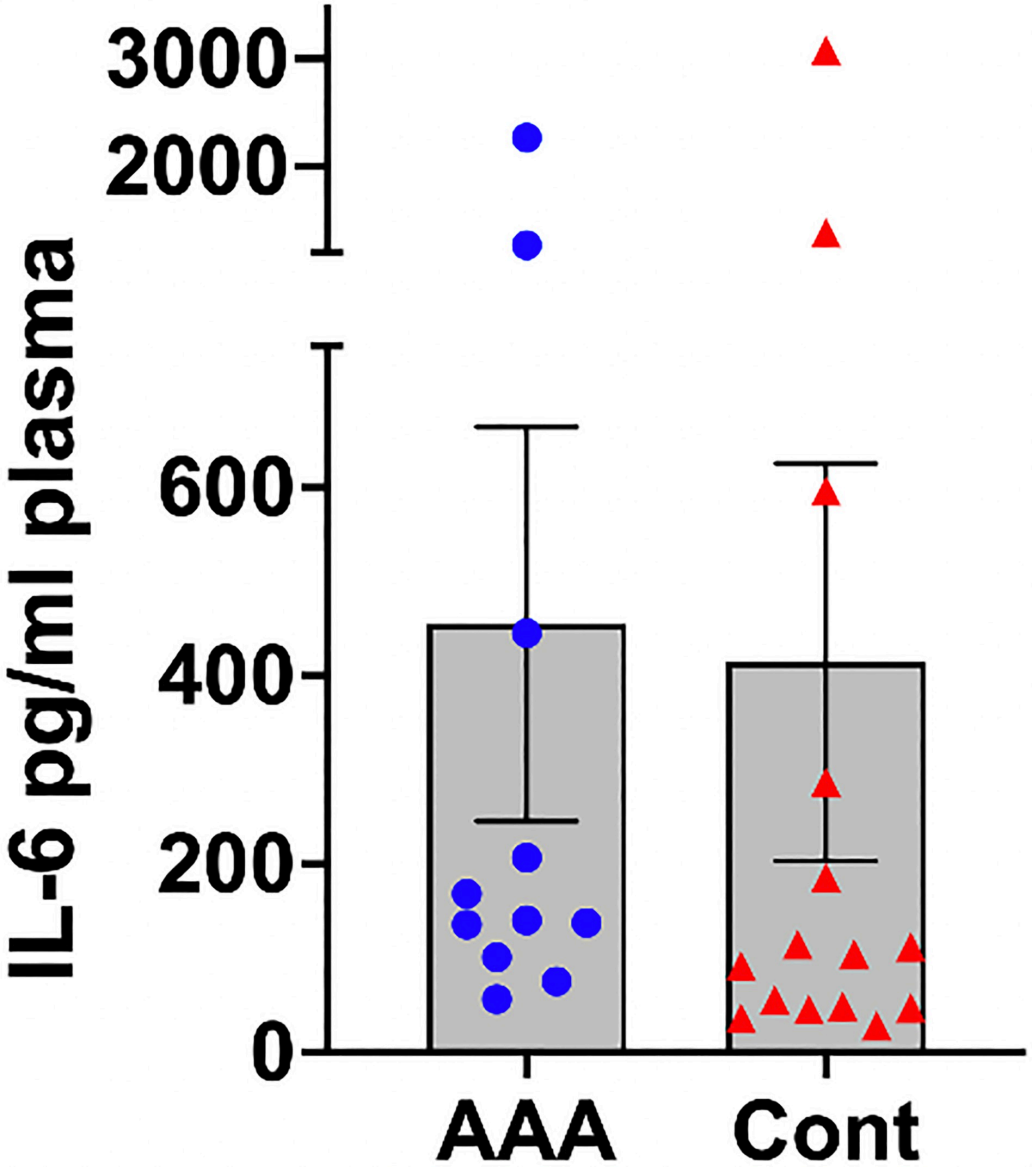
Interleukin-6 levels in the control and AAA groups. There was no significant difference in plasma levels of IL-6 between groups (*P* = .17). AAA, abdominal aortic aneurysm; IL-6, interleukin-6.

Quantitative Real-Time PCR (qRT-PCR) was used to validate the exosomal expression levels of miR-122-5p in all 24 patients (S1 Data). The mean relative expression of miR-122-5p was significantly decreased in the AAA group compared to the control group, which was consistent with the results from small RNA sequencing (2.38 vs 8.85, *P* = 0.026) (Fig 4) (S2 Data). There was no statistically significant difference in the median levels of IL-6, between the AAA and control groups (140 pg/ml [IQR 100 pg/ml to 445 pg/ml] vs. 104 [IQR 46.8 to 287.0], *P* = .17) (Fig 5).

## Discussion

The burden of AAA disease is widely recognized, but the current method for population-based screening, duplex US, requires significant resources and is not uniformly available. This is particularly important given the long latency period between the ability to detect AAA disease and the need for treatment. The identification of a biomarker to screen or monitor disease progression in AAA would allow for the development of cost-effective and blood-based screening tests and potentially laboratory-based surveillance of disease progression. Circulating exosomal miRNAs are promising molecules with the potential to be used as biomarkers.

In an attempt to discover novel biomarkers of AAA disease, we used NGS to characterize and compare the plasma exosome miRNA profiles of patients with AAA and non-aneurysmal controls. Our analysis showed that 40 miRNAs were differentially expressed in plasma exosomes from AAA patients versus non-aneurysmal controls. The most notable finding was that patients with AAA had significantly less miRNA 122-5p expression in circulating plasma exosomes compared to control patients, a result confirmed by qRT-PCR of the exosome fraction. Consistent with the plasma results of the current study, one of the few studies that evaluated the miRNA expression profile of aortic tissue from aneurysms identified miRNA-122-5p as one of six miRNAs that was downregulated in both small and large aneurysms compared to control tissue [31].

Our finding of decreased exosomal miR-122 expression in AAA patients may be closely linked to the role miR-122 plays in regulating extracellular matrix (ECM) processing by matrix metalloproteinases (MMPs) [32]. Network analyses demonstrated that several MMPs are potential targets of miR-122-5p, with MMP2 and MMP7 being ranked the highest. MMP2 and MMP7 have been shown to play a critical role in the pathophysiology of AAA and cellular signaling leading to ECM breakdown [33, 34]. In addition to network pathway analyses in this study demonstrating that miR-122-5p may regulate several matrix metalloproteinases implicated in the pathogenesis of AAA, multiple studies have confirmed both MMP-2 and MMP-9 as targets of miR-122-5p [33, 34]. These findings initially came from studies of cancer progression, where decreased miR-122-5p leads to increased MMPs and increased metastatic potential [34, 35]. More recently, several studies have shown an association of elevated miR-122-5p with obesity and insulin resistance [36]. In fact, this association appears to result in the increased cardiovascular fibrosis seen in diabetes, with a very recent study of diabetic cardiomyopathy demonstrating that miRNA 122-5p was increased in heart failure patients with diabetes compared to age- and disease-matched controls. Further, this investigation demonstrated miR-122-5p directly binds MMP-2 mRNA and that this leads to increased cardiac stiffness in mice [37]. MMP 2 and 9 have long been known to be critical in the progression of AAA through the breakdown of ECM, in some ways representing the opposite histopathologic change to that of diabetic fibrosis [38]. Interestingly, meta-analyses have shown that individuals with diabetes mellitus have reduced risk of AAA progression, although the rationale has remained elusive [39]. MicroRNA 122-5p, as a potential master regulator of MMPs, may be the molecular connection between the relationship of diabetes and AAA.

Conversely to our findings, miR-122-5p was significantly upregulated in patients with hyperlipidemia and closely associated with both the presence and severity of coronary heart disease [40]. In addition, miR-122 was increased in the setting of both stable and unstable angina [41]. This may be in part due to its ability to exert both pro and anti-inflammatory regulatory effects in cardiovascular inflammation [42, 43]. Vascular inflammation is central to the pathogenesis of AAA. AAA formation is initiated by an influx of immune cells that secrete inflammatory cytokines (TNF-α, IL-1β, IL-6, MCP-1) and activate metalloproteinases, resulting in smooth muscle apoptosis, extracellular matrix degradation, and elastin fragmentation [44, 45]. In fact, recently miR-122-5p has recently been identified to play a critical role in the formation of thoracic aortic aneurysms in a mouse model [32]. In this model, miR-122-5p was shown to regulate inflammatory cytokines and matrix remodeling within the aortic wall. The function and specific role of miR-122 in AAA inflammation is still largely unknown, but given the aforementioned study findings, one could hypothesize that reduced levels of exosomal miR-122 lead to an upregulation of proinflammatory mediators, which in turn promote the inflammatory microenvironment responsible for chronic inflammation and progression of AAA. These hypotheses warrant further investigations and could aid in our understanding of the function of miR-122 in AAA.

### Limitations

The greatest limitation to this study is the difference in comorbidities and medication use between the two groups. Patients with AAA were more likely to have CAD and more likely to be taking aspirin and statins. These comorbidities and medications may alter miRNA signatures that could interfere with the attempt to identify biomarkers specific to AAA. Particularly aspirin, which has been shown to modulate exosomal cargo [46]. However, given the small sample sizes and exploratory nature of this study it was not appropriate or feasible to attempt to account for these differences between groups with a regression analysis, which would have been susceptible to overcorrection. Although challenging given the comorbid nature of AAA with other atherosclerotic diseases, future studies should attempt to have at least similar rates of ASA and statin use between groups. In addition, the small sample size may have had an influence on the number of miRNAs that were significant after adjustment for the false discovery rate. Future studies will need to evaluate the significance of miRNA 122 and other potential markers in more patients to validate our findings and ensure other variables are not responsible for these differences. Finally, the majority of participants were male; future studies will need to account for any differences in miRNA expression based on gender.

## Conclusion

Our analysis identified several differentially expressed miRNAs between patients with AAA and control patients, with AAA patients having significantly reduced levels of miR-122-5p in plasma exosomes. This is a novel exosome-associated miRNA previously unreported in the setting of AAA and thus warrants further investigation to determine both its use as a diagnostic biomarker and potential implications in AAA pathogenesis.

## Supporting information

S1 Fig Read-length distribution of exosomal miRNA (**A**). Each colored line represents an individual sample from either control or AAA group. Nt, nucleotides. Relative distribution of exosomal RNA biotypes in all the samples based on the number of normalized reads (**B**). Protein coding and miRNA were the most represented biotypes in the samples.
(TIFF)

S1 Data qRT-PCR results. (XLSX)

S2 Data miRNA differential expression. (XLSX)

S1 Raw Image (TIFF)
