# Supplementary figures and images for: Patients with abdominal aortic aneurysms have reduced levels of microRNA 122-5p in circulating exosomes
Source: PLoS One. 2023 Feb 14;18(2):e0281371. doi: 10.1371/journal.pone.0281371 (PMC9928131; doi:10.1371/journal.pone.0281371)

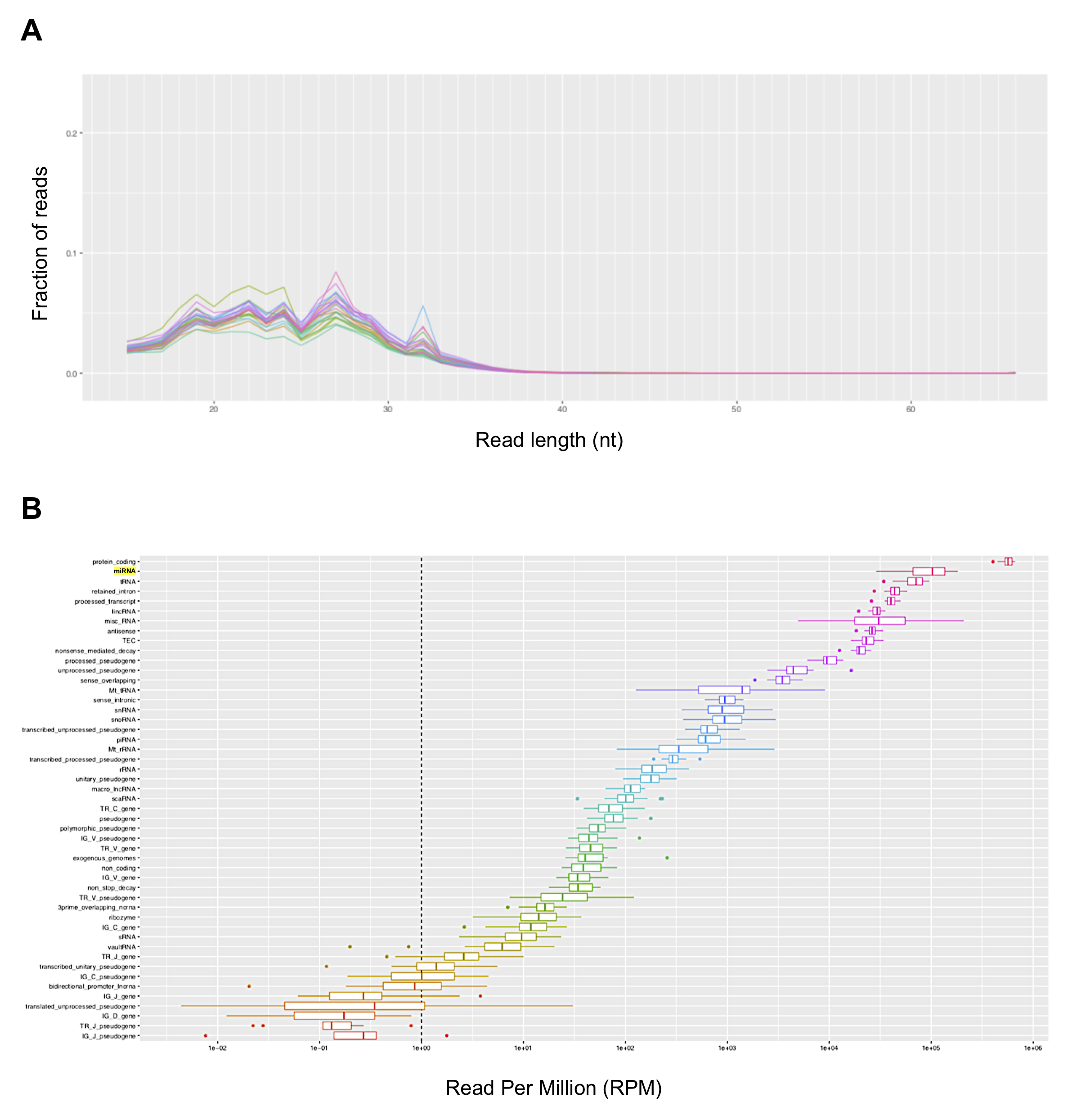

Supplement: S1 Fig — Read-length distribution of exosomal miRNA (A). Each colored line represents an individual sample from either control or AAA group. Nt, nucleotides. Relative distribution of exosomal RNA biotypes in all the samples based on the number of normalized reads (B). Protein coding and miRNA were the most represented biotypes in the samples. (TIFF) [file pone.0281371.s001.tiff]

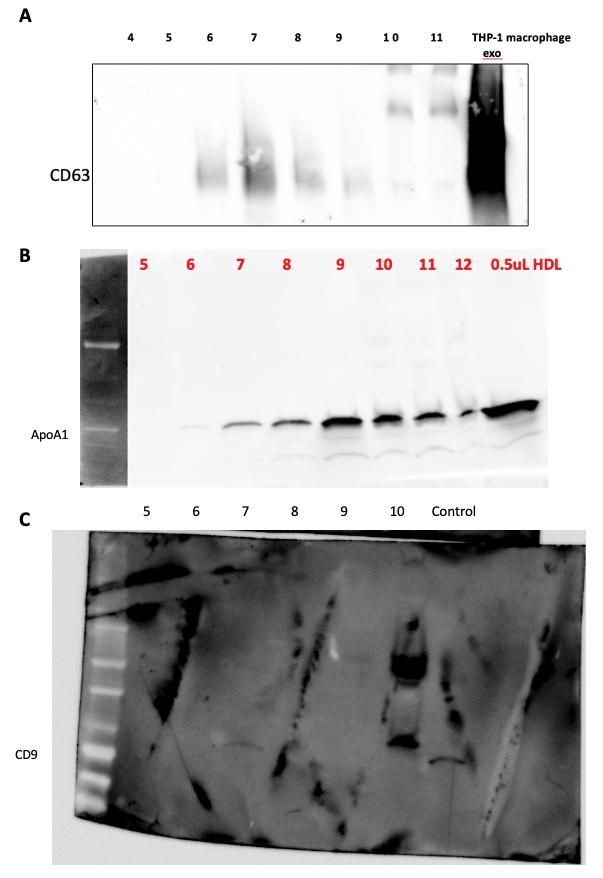

Supplement: S1 Raw Image — (TIFF) [file pone.0281371.s004.tiff]
